# Diverging pH dependence and photocycle dynamics across members of the CryoRhodopsin clade

**DOI:** 10.1016/j.bpj.2026.01.031

**Published:** 2026-01-23

**Authors:** Sarah Warrelmann, Gerrit H.U. Lamm, Kirill Kovalev, Josef Wachtveitl

**Affiliations:** 1 Institute for Physical and Theoretical Chemistry, Goethe University Frankfurt, 60438 Frankfurt am Main, Germany; 2 European Molecular Biology Laboratory, EMBL Hamburg c/o DESY, 22607 Hamburg, Germany

## Abstract

A new clade of microbial rhodopsins (MRs) called CryoRhodopsins (CryoRs) was recently discovered and characterized to possess a near-UV-absorbing intermediate populated for minutes at neutral to alkaline conditions. A detailed characterization of one member, CryoR1, showed a strong pH dependence, causing a hindered retinal isomerization and a significant acceleration of the photocycle dynamics at acidic conditions. Here, we present a spectroscopic study on the pH dependence of four other CryoRs (CryoR2–5), revealing strongly deviating photophysical properties within this subfamily under acidic conditions. Although all investigated CryoRs possess a minute-long photocycle duration at neutral to alkaline conditions, acidification shortens the photocycle to seconds and leads to variation in the observed photocycle schemes. Furthermore, this is not directly reflected in the absorption spectra and initial photoreactions, since CryoR4 and CryoR5 show very weak pH dependence, whereas CryoR2 and CryoR3 do show pH-dependent shifts. In addition, CryoR3 is unique within both CryoRs and MRs in general, since a near-UV-absorbing species is clearly present throughout the whole pH range in the dark spectrum. The observed diversity suggests strong mechanistic divergence within this clade of MRs, a feature that was not observed for other clades of MRs. It also further supports functional relevance of the long-living near-UV-absorbing state of CryoRs under neutral and alkaline conditions.

## Significance

Within a clade of microbial rhodopsins, photoswitching and photocycle mechanisms are expected to have comparable features due to high sequence homology and structural similarity. This study shows that each investigated member of the newly discovered clade CryoRhodopsins (CryoRs) has a unique photocycle with different intermediates on separate timescales. Moreover, two members show pH-independent absorption spectra and initial photoreactions, whereas a third one shows a dominant deprotonated species that directly populates the ground state upon illumination. Understanding their functional diversity helps to pinpoint the role of certain residues in pH dependence and general photocycle dynamics within the CryoR family.

## Introduction

Microbial rhodopsins (MRs) comprise one of the most abundant families of photoreceptors, who convert solar into biochemical energy, thus being fundamentally important for life on earth. Their phylogenetic tree is continuously expanding due to metagenomic analysis and molecular phylogenetic methods (1,2). A new clade of MRs termed CryoRhodopsins (CryoRs) was recently discovered by bioinformatic search (3). Seven-letter motifs corresponding to T46, R82, D85, T89, D96, D212, and K216 of the prototypical MR bacteriorhodopsin (BR) were analyzed, and the unusual motif pattern RR XXX DK, characterized by the arginine at the position of T46, which clustered together in the phylogenetic tree, was identified as a new clade, with the prominent shared feature of a very slow photocycle at neutral pH dominated by the blue-shifted M intermediate. Indeed, their unique R57 (numbering of the most characterized CryoR1) arginine residue was found to be responsible for the unusually slow photocycles. The long-living M state also prompted the hypothesis that their biological role might be a photosensory one. The characterization of such MRs is essential for fundamental scientific knowledge, since it provides access to novel molecular mechanisms of ion transport in different directions (4,5,6,7,8), ion translocation (9,10), light sensing, and signal transduction (11). Research efforts were further strengthened by the evolving field of optogenetics (12,13), which utilizes MRs to manipulate membrane potentials in living organisms (14), and control pH of the cytosol and intracellular organelles (15) in response to light stimuli. Detailed knowledge about the various molecular pathways and protein functionality eventually resulted in nonnative protein variants with modified characteristics (16,17) and, consequently, improved or expanded applicability in optogenetics. Thus, the continuous expansion of this toolbox is of considerable scientific interest.

MRs exist in a variety of colors, with their absorption maximum mostly being found between 500 and 600 nm. The exact position is sensitive to many factors such as the position of the counterion(s) and their protonation state. The photocycle is generally initiated by photoisomerization of the protonated chromophore by excitation into the absorption maximum from all-*trans* to 13-*cis*-retinal. Subsequently, the protein undergoes a series of photointermediates, starting with the vibrationally excited J intermediate followed by the K intermediate, containing twisted retinal. The proton transfer from the retinal Schiff base (RSB) to the primary proton acceptor leads to the M intermediate, which is spectrally blue-shifted due to the deprotonated 13-*cis*-retinal. Afterward, the RSB is reprotonated by a carboxylic proton donor, and the chromophore reisomerizes back to the dark state. Many photocycles contain additional intermediates L, N, and O, which are representative of precursor states, changes in the protein backbone conformation, and temporally separated reprotonation, reisomerization, and structural reorganization steps (18).

**Figure 1:**
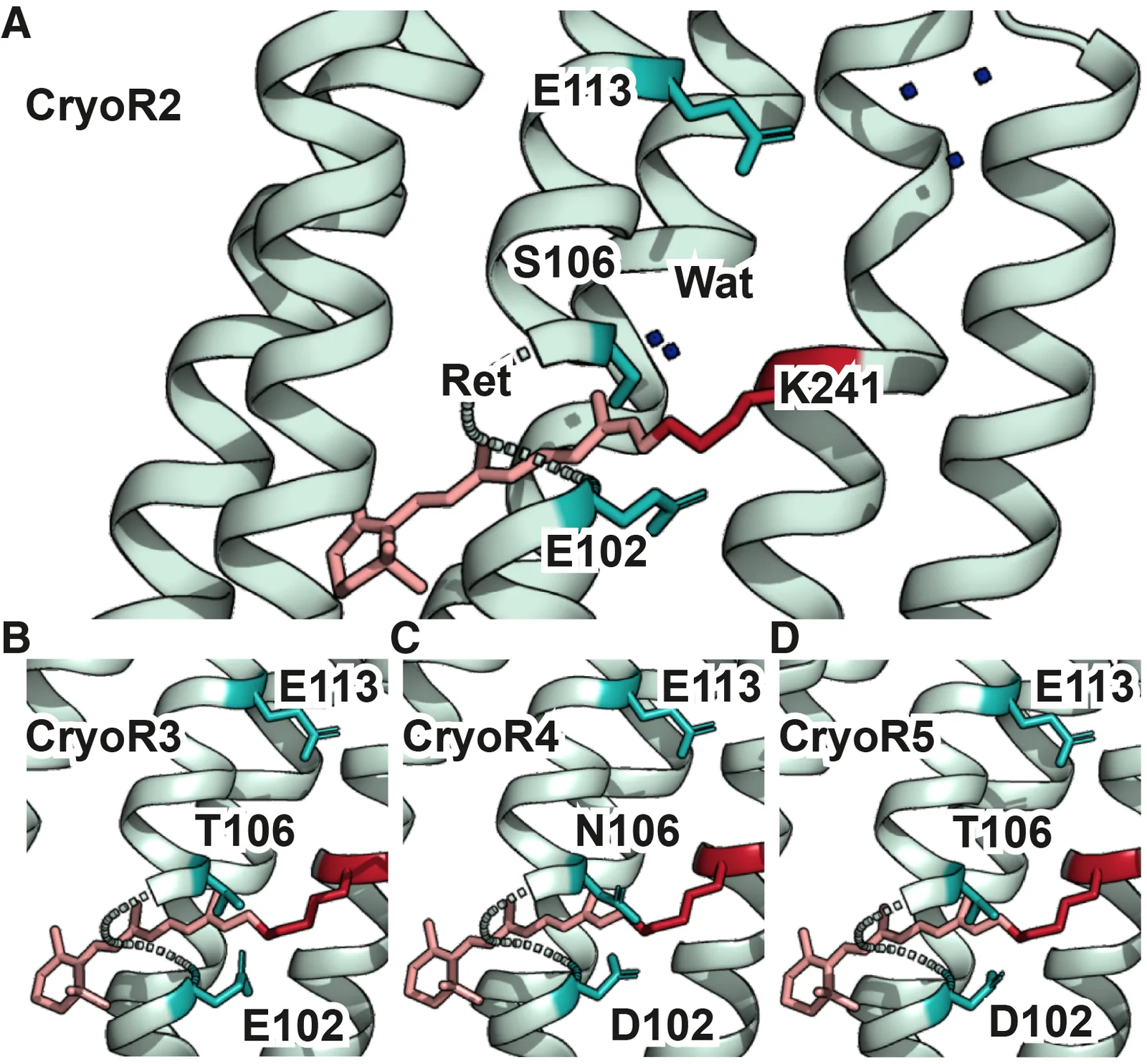
Structural representation of CryoR2, CryoR3, CryoR4, and CryoR5. (*A*) RSB region of CryoR2 (PDB: 8R0P (3)). The motif is colored in light blue, the RSB K241 in red, and the retinal is rose colored. Blue dots indicate water molecules. Predictions for CryoR3 (*B*), CryoR4 (*C*), and CryoR5 (*D*) are only labeled where their motif differs. Structures of CryoR3-5 were predicted using AlphaFold3 (21,22). All structural visualizations were created using PyMOL software (23).

CryoR1 (RR EAE DK motif) was extensively analyzed by Lamm et al. (3), yielding unique spectral behavior, like a strong pH dependence of its absorption spectra and kinetics and a hindered retinal isomerization at acidic pH conditions. From the literature, it is known that members of the same clade are generally expected to have similar patterns in their dynamic behavior (19,20), which is surprisingly not the case for the CryoRs. Although all studied members of the clade have a slow photocycle, they differ in other photophysical and general properties, prompting further investigation on all timescales from femtoseconds to minutes (steady state). The three-letter-motifs of the members investigated here are ESE (CryoR2), ETE (CryoR3), DNE (CryoR4), and DTE (CryoR5) (Fig. 1), variating between glutamic and aspartic acid at position E102 and the different hydroxylic or an amide residue at position 106. Due to the strong pH dependence of the main absorption band and photocycle in the case of CryoR1, different pH conditions from acidic to alkaline were investigated for CryoR2–5. Our study showed that CryoR1-like behavior is not conserved within the clade. Furthermore, it was expected from the literature that most MRs lose their functionality in extremely acidic conditions (24,25,26,27,28), which is likely only partly the case for CryoRs. The diverse response of CryoRs to pH changes further supports that the few differences between the CryoRs in their amino acid sequence have substantial effects on their dynamics and functionality. Analysis of their characteristics and the underlying mechanisms might facilitate a better understanding of rhodopsin dynamics as a whole, particularly the pH independence of CryoR4 and CryoR5 in contrast to the pH-dependent behavior of CryoR2 and CryoR3.

## Materials and Methods

### Protein expression, solubilization, and purification

Proteins were prepared as described in Lamm et al. (3). *E. coli* cells, strain C41(DE3), were transformed with pET15b plasmid containing the gene of interest. Transformed cells were grown at 37°C in shaking baffled flasks in an autoinducing medium ZYP-5052 (29), containing 10 mg/L ampicillin. They were induced at an OD_600_ of 0.8–0.9 with 1 mM isopropyl-β-D-thiogalactopyranoside (IPTG). Subsequently, 10 μM all-*trans*-retinal was added. Incubation continued for 3 h. The cells were collected by centrifugation at 4000 × *g* for 25 min. Collected cells were disrupted in an M-110P Lab Homogenizer (Microfluidics, Westwood, MA, USA) at 25,000 psi in a buffer containing 20 mM Tris-HCl (pH 8.0), 5% glycerol, 0.5% Triton X-100 (Sigma-Aldrich, St. Louis, MO, USA), and 50 mg/L DNase I (Sigma-Aldrich, St. Louis, MO, USA). The membrane fraction of the cell lysate was isolated by ultracentrifugation at 125,000 × *g* for 1 h at 4°C. The pellet was resuspended in a buffer containing 20 mM Tris-HCl (pH 8.0), 0.2 M NaCl, and 1% DDM (Anatrace, Affymetrix, Santa Clara, CA, USA) and stirred overnight for solubilization. The insoluble fraction was removed by ultracentrifugation at 125,000 × *g* for 1 h at 4°C. The supernatant was loaded on a Ni-NTA column (Qiagen, Venlo, Netherlands), and the protein was eluted in a buffer containing 20 mM Tris-HCl (pH 8.0), 0.2 M NaCl, 0.4 M imidazole, and 0.1% DDM. The eluate was subjected to size exclusion chromatography on a Superdex 200i 300/10 (GE Healthcare Life Sciences, Chicago, IL, USA) in a buffer containing 20 mM Tris-HCl (pH 8.0), 100 mM NaCl, and 0.03% DDM. In the end, protein was concentrated to 70 mg/mL and stored at −80°C.

### Sample preparation

Experiments at pH 3.5 were performed in a 20 mM sodium citrate buffer. At pH 5.5, a 20 mM MES buffer was used. For pH 8.0, a 20 mM Tris buffer was prepared. At pH 10.5, the buffer consisted of 20 mM CAPS. All buffers additionally contained 200 mM NaCl and 0.05% DDM. The protein stock solution was adjusted to the respective conditions by washing it three times with the respective buffer. The samples and cuvettes were always stored and prepared in the dark.

### Steady-state absorption spectroscopy and illumination experiments

All absorption spectra were recorded on an absorption spectrometer (Specord S600, Analytik Jena, Jena, Germany). Measurements were performed at room temperature. Illumination and pH titration experiments were conducted in a 2 × 10 mm cuvette. Each sample was illuminated for 100 s through the 2-mm side with a LED at the respective wavelength (Table 1) to reach the photostationary state (PSS).

**Table 1:** LEDs and the respective power used for illumination

	LED	Power
CryoR2 pH 3.5	M625L3	20 mW
CryoR2 pH 10.5	M530L3	10 mW
CryoR3	M505L4	10 mW
CryoR4	M590L3	10 mW
CryoR5	M565L2	10 mW
Back illumination	M420L2	10 mW

All LEDs are by Thorlabs (Newton, NJ, USA).

### Ultrafast transient absorption spectroscopy

Ultrafast transient absorption (TA) spectroscopy experiments were performed using two equivalent home-built pump-probe setups described in detail elsewhere (30,31,32). A Ti:Sa chirped pulse regenerative amplifier (MXR-CPA-iSeries, Clark-MXR, Dexter, MI, USA) served as the fs-laser source for the setup used in the CryoR2 measurements. It was operated at a central wavelength of 775 nm and a repetition rate of 1 kHz, resulting in laser pulses with a pulse width of 150 fs. Excitation pulses were generated from a home-built two-stage noncollinear optical parametric amplifier (NOPA) with an interlaid prism compressor. For probing absorbance changes, the laser fundamental was focused into a 5-mm CaF_2_ crystal to generate supercontinuum pulses. The probe light was then divided and guided through sample and reference paths. Two identical spectrographs (Multimode, AMKO, Tornesch, Germany), equipped with a grating (500 nm blaze, 1200 grooves per mm), a photodiode array (S8865-64, Hamamatsu Photonics, Shizuoka, Japan), and a driver circuit (C9918, Hamamatsu Photonics, Shizuoka, Japan) were used for signal detection. The obtained signals were digitized via a 16-bit data acquisition card (NI-PCI-6110, National Instruments, Austin, TX, USA).

The second fs-laser system contains an amplifier (Spitfire Ace-100F-1K, Spectra-Physics, Milpitas, CA, USA) that is seeded by a Ti:Sa oscillator (Mai Tai SP-NSI, Spectra-Physics, Milpitas, CA USA) and pumped by a Nd:YLF laser (Empower 45, Spectra-Physics, Milpitas, CA, USA). It was operated at a central wavelength of 800 nm emitting 100-fs pulses with a 1-kHz repetition rate. Excitation pulses were generated in a home-built two-stage NOPA with an interlaid prism compressor and spectrally adjusted according to the sample absorption maxima. White light super continuum generation was achieved by focusing the laser fundamental into a CaF_2_ crystal (3 mm). The signal was detected using a custom spectrometer containing a sensor (MMS UV-VIS II, Carl Zeiss, Oberkochen, Germany) and preamplifying electronics (Tec5, Steinbach, Germany).

Generally, the pump wavelength was adjusted to the respective absorption maximum, and pump and probe pulses were set to the magic angle (54.7°) configuration to eliminate anisotropic effects. Additionally, the sample was constantly moved in a plane perpendicular to the excitation beam to avoid multifold excitation and sample degradation. Samples were constantly illuminated with a LED (M405L4 (CryoR2), M420L2 (CryoR3-5), Thorlabs, Newton, NJ, USA), operated at 5 mW, to regenerate equal starting conditions for the start of each scan. Absorption spectra were taken before and after each experiment to verify sample quality.

### Transient flash photolysis spectroscopy

The excitation pulse was generated by an ns Nd:YAG laser (SpitLight 600, Innolas Laser, Krailling, Germany), which pumps an optical parametric oscillator (OPO, preciScan, GWU-Lasertechnik, Erftstadt, Germany). The OPO was set to generate pump pulses at the respective maximum absorption with an average pulse energy of ∼2.2 mJ/cm^2^. The probe light was provided by a Xenon or a Mercury-Xenon lamp (LC-8, Hamamatsu Photonics, Shizuoka, Japan) and guided through two identical monochromators (1200 L/mm, 500 nm blaze), one in front of and the other after the sample. Absorption changes were detected with a photomultiplier tube (Photosensor H6780-02, Hamamatsu Photonics, Shizuoka, Japan) and converted to an electric signal, which was recorded using two oscilloscopes (PicoScope 5244B/D, Pico Technology, Cambridgeshire, England) with overlapping timescales. For each transient, 30 acquisitions were measured and averaged to improve the S/N ratio. As for minutes-long transients, less probe light was used to minimize actinic effects, the S/N ratio is smaller in the respective measurements. To obtain data files with a reasonable size for further analysis, raw data files were reduced using forward averaging and a combined linear and logarithmic timescale. Detergent-solubilized samples were measured in a 2 × 10 mm quartz cuvette and prepared to have a protein concentration equal to an optical density of 1.0 at an optical pathlength of 10 mm. Absorption spectra were taken before and after each experiment to verify sample quality.

### Analysis of time-resolved spectroscopic data

Analysis of time-resolved spectroscopic data was performed using the OPTIMUS software (33). The data of the ultrafast TA and transient flash photolysis measurements were subjected to the model-free lifetime distribution analysis, yielding the lifetime distributions of the individual photointermediate transitions, which are summarized in a lifetime density map (LDM). The mentioned lifetimes of the photointermediate transitions were obtained from the point with maximum amplitude of the respective lifetime distribution.

## Results

### pH-dependent steady-state properties

To gain insights into the photophysical properties of CryoR2–5 and their response to changes in ambient pH, the steady-state absorption properties were investigated in a pH-dependent manner. Our data elucidates varying pH-dependent properties of the studied CryoRs.

**Figure 2:**
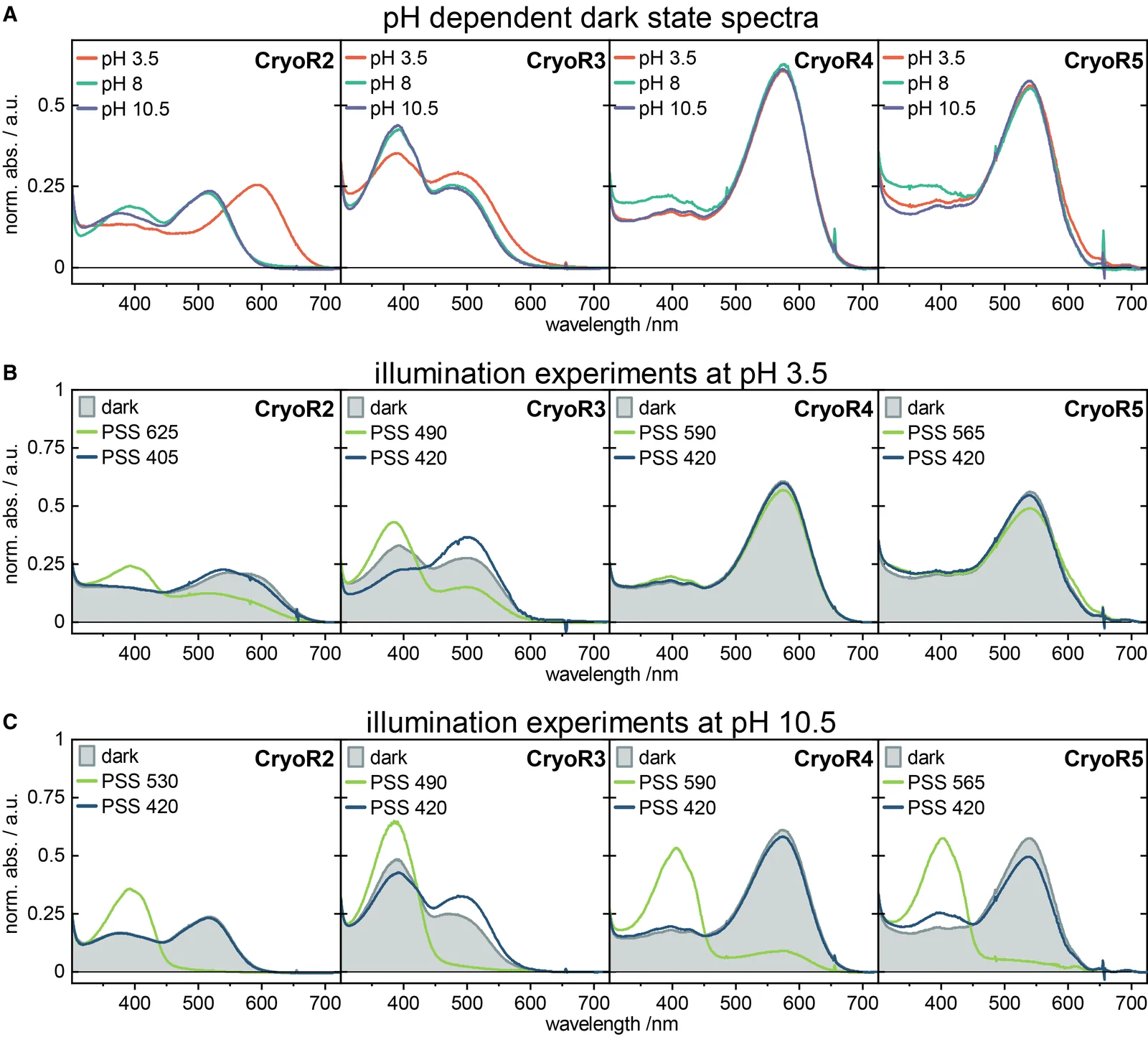
pH-dependent absorption spectra of CryoRs and illumination experiments at acidic and alkaline conditions. The top panels (*A*) show the dark state spectra of the investigated CryoR2, CryoR3, CryoR4, and CryoR5 at different pH values (*red, pH 3.5; green, pH 8; blue, pH 10.5*). The middle (*B*) and bottom (*C*) panels show the dark-state spectra (*gray area*), as well as the PSS spectra after illumination of the main absorption band (*green line*) and the spectra after illumination of the obtained PSS with blue light (*blue line*) to recover the dark state of each investigated CryoR at pH 3.5 (*middle*) and 10.5 (*bottom*). All spectra are normalized to the protein peak at 280 nm.

Like CryoR1 (3), CryoR2 shows an 80-nm spectral shift upon pH change (Fig. 2 *A*) from acidic to alkaline conditions. The spectral blue shift is accompanied by the rise of an absorption band at 400 nm indicating a species with a deprotonated RSB. CryoR3, in contrast, does not show a pronounced fine structure, instead having two similarly strong maxima at 380 and 490 nm. The strong blue maximum reflects a considerable amount of deprotonated RSB even in the ground state (GS). At pH 3.5, the states associated with the two maxima are almost equally populated, whereas at high pH values the deprotonated state dominates. pH changes do not lead to a gradual wavelength shift but merely alter the population of the two distinct states correlated with absorption maxima. Both CryoR4 and CryoR5 show no spectral adaptation upon pH change. This suggests their structural stability, with polar groups in the retinal binding pocket (RBP) likely being shielded from the bulk. CryoR4 displays one distinct maximum at 575 nm and a weak band with a fine structure at around 400 nm across the whole pH range. In comparison, CryoR5 has a blue-shifted maximum at 540 nm, but otherwise, it displays exactly the same features as CryoR4 with a weak band with a fine structure around 400 nm. Such a three-partite fine structure was observed in the past for the M state of xenorhodopsins (34,35,36,37) and this spectral shape was assigned to originate from an enforced planarity of the retinal chromophore (38,39). Hence, we suggest a similar planar retinal structure for CryoR4 and CryoR5, which could be confirmed in future FTIR measurements in the hydrogen-out-of-plane region. It is conserved over the whole pH range for both CryoRs, further demonstrating their consistency upon pH change.

In a similar manner to CryoR1, illumination at the respective wavelength of the absorbance maxima causes efficient GS depopulation accompanied by the formation of a blue-shifted M-like state for all CryoRs at neutral and alkaline pH values (Fig. 2 *C*). Due to the long lifetime of the CryoR M state, we assume that we see its accumulation even in steady state under continuous illumination, as well as similar decay times once the light has been switched off (Fig. S2). The GS of Cryo R2 can be depopulated with the rise of the near-UV-absorbing state at acidic, neutral, and alkaline pH. In line with the shortened lifetime of the near-UV-absorbing M state of CryoR2 at low pH reported earlier, deprotonation of the GS at pH 3.5 is incomplete, whereas it can be fully depopulated at pH 10.5 (Fig. 2 *B*). Noteworthy, similar strong depopulation of the GS state by illumination at the wavelengths of the respective maxima for CryoR4 and CryoR5 is only possible at neutral and alkaline pH values, whereas no near-UV-absorbing state can be accumulated at acidic pH. This is likely due to photocycle intermediates being too short-lived for accumulation at room temperature (described in more detail later in the manuscript). The fine structure of the M state of CryoR4 and CryoR5 most likely indicates a retro-retinyl like spectral shape associated with a planarized retinal conformation (38,39). The dark state spectra of all studied CryoRs can be recovered by illumination of the PSS with a 420-nm LED by the blue light quenching effect, which is well known for many MRs including CryoRs (39,40,41,42).

A special behavior was observed for CryoR3. As reported before, green light illumination yields an increased absorbance at 400 nm across all pH values (Fig. 2 *B* (pH 3.5) and Fig. 2 *C* (pH 10.5)), with a stronger signal difference at pH 10.5, where the GS absorbance can be reduced to zero. On the other hand, blue light illumination of CryoR3 does not solely repopulate the dark state spectrum but also leads to a stronger absorbance at 490 nm than the initial dark state. Illumination with blue light of the dark state also yields an increased GS absorbance at 500 nm (Fig. S3). Therefore the GS of CryoR3 might be heterogeneous with at least two substates that interconvert upon illumination.

### Diversity of ultrafast dynamics

To uncover the temporal evolution of the CryoRs upon illumination, their behavior was monitored using time-resolved femtosecond spectroscopy, starting with the retinal isomerization as the initial photoreaction, since CryoR1 already showed very unusual behavior compared with the majority of MRs.

**Figure 3:**
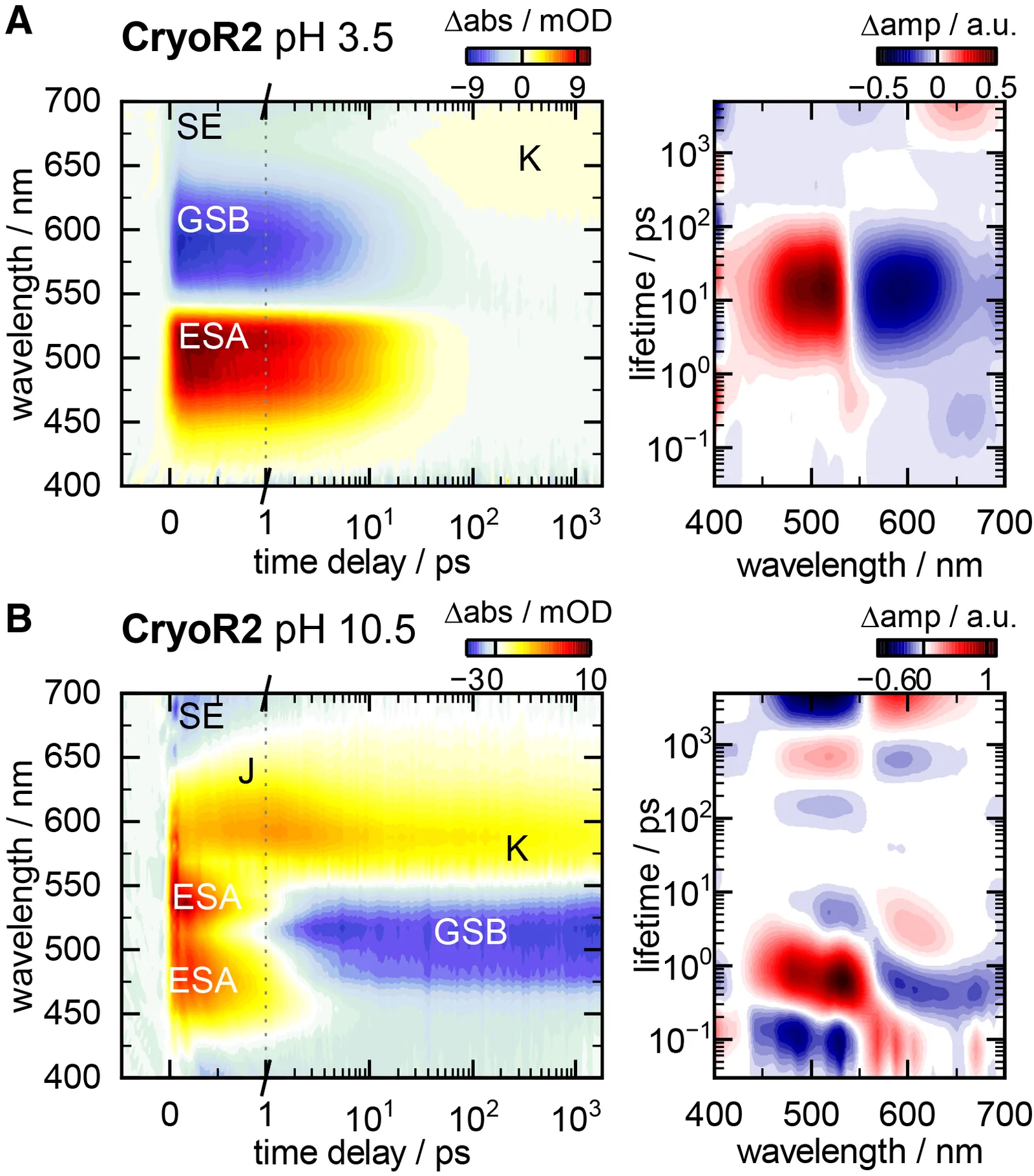
Ultrafast TA measurement of CryoR2 at pH 3.5 and pH 10.5. CryoR2 ultrafast map and LDM at pH 3.5 (*A*) and pH 10.5 (*B*). The signal amplitude is color coded as follows: positive (*red*), none (*white*), and negative (*blue*) difference absorbance. The x-axis is linear until 1 ps and logarithmic afterward. The corresponding LDMs illustrate the kinetic components of the respective measurement.

The pH dependence of CryoR2 absorption is also reflected in its ultrafast dynamics (Fig. 3). It shows a strong pH dependence as the efficiency of the initial photoreaction decreases with acidification and the intermediates are red-shifted as expected from the absorption spectrum. At acidic pH, the excited state absorption (ESA) is long lived (∼14 ps), and a faint K-intermediate signal rising in parallel with the decay of the ESA can be observed. The measurement at pH 10.5 shows two ESA signals, which opens up two possibilities: either one very broad ESA is split in two by the overlapping ground state bleach (GSB), or a second excited state (ES) is involved, which is more likely as the signal shape of the ESA would be reflected in the signal shape of the GSB otherwise. At alkaline pH, in addition to a general acceleration of the ES decay, strong J and K intermediates are formed, indicating a larger fraction of proteins entering the photocycle. To better compare the measurements between the two pH values and remove differences naturally introduced by the experiment, we calculated the ratios of the signal intensities for the ESA at 80 fs after excitation (originating from the excited sample) and the GSB at 500 ps after excitation (originating from the fraction actually entering the photocycle) (Table S1). As a benchmark, the well-characterized sodium pump KR2 (26) is used, whose ratio increases from 12% to 45% at more alkaline pH values. Since these values are specific for each protein, only the order of magnitude should be considered. CryoR2 increases from 2% to 26% and therefore follows the general pattern of typical MR ultrafast dynamics (24,25,26,27,28). However, CryoR3–5 deviate from this classical behavior.

**Figure 4:**
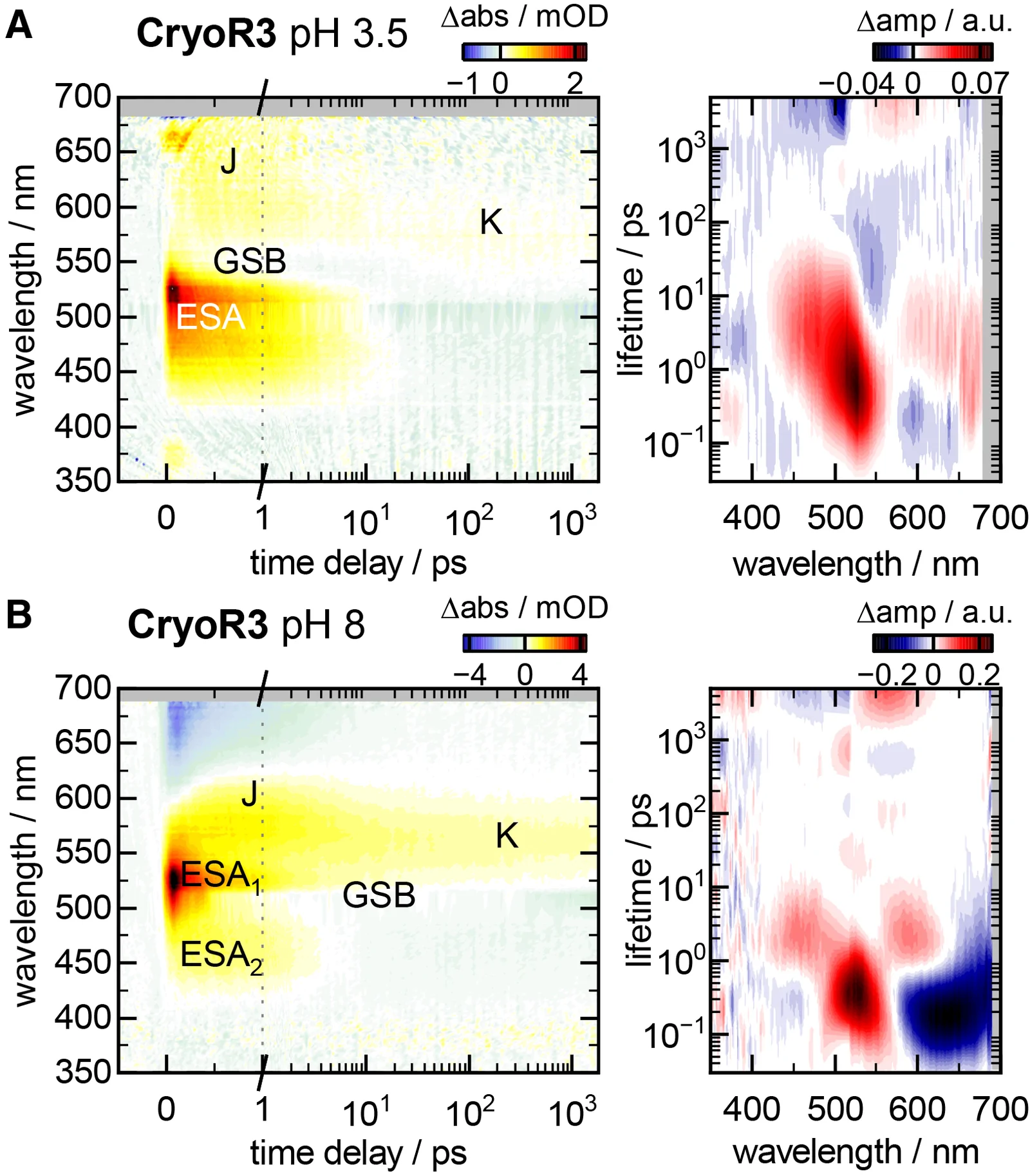
Ultrafast TA measurement of CryoR3 at pH 3.5 and pH 8. CryoR3 ultrafast map and LDM at pH 3.5 (*A*) and pH 8 (*B*). The signal amplitude is color coded as follows: positive (*red*), none (*white*), and negative (*blue*) difference absorbance. The x-axis is linear until 1 ps and logarithmic afterward. The corresponding LDMs illustrate the kinetic components of the respective measurement.

CryoR3 most resembles the ultrafast kinetics of channelrhodopsin-2 (40,43) and VirChR1 (44). Their ultrafast kinetics seem to be shared among rhodopsins with an absorption maximum near 500 nm. At acidic pH (Fig. 4 *A*), the GSB signal is barely visible, and the intensities of the J and K states are very weak, indicating a very small share of proteins following a reactive pathway. The stimulated emission (SE) is not observed in the accessible spectral window, and the photocycle intermediates are red-shifted by 50 nm. The J state decays at ∼1 ps similar to other MRs. The ESA signal, which strongly overlaps with that of the GSB, decays multiexponentially, with the red-shifted signal region decaying earlier and the blue-shifted one decaying for ∼10 ps. At neutral pH (Fig. 4 *B*), almost all signals up to ∼5 ps are superimposed, as both the J and K intermediates are blue-shifted compared with pH 3.5. A major part of the ESA signal decays at ∼0.3 ps together with the SE at 675 nm. The rise of the J state also occurs below 1 ps. At ∼2 ps, the J-to-K transition occurs as indicated by the positive amplitude in the LDM (550–650 nm). At the same time, a positive signal potentially corresponding to the second ESA below the GSB centered at 460 nm decays. This simultaneous decay of ESA and rise of the K intermediate matches temporally with the ESA decay at pH 3.5, which suggests mixed kinetics between less efficient low pH and more efficient high pH kinetics. The K intermediate and the remaining GSB signal are visible until the end of the experimentally available timescale. The calculated GSB/ESA ratios at both pH values did not show significant differences (Table S1), but as expected from the absorption spectra, the position of spectral signals and lifetimes of the respective states of CryoR3 are strongly influenced by pH changes. It has to be noted that the sample was continuously illuminated with 420-nm light due to the long photocycle duration. Thus, we cannot rule out that this illumination influenced the kinetics of the sample due to the significant fraction of the GS species in the near-UV-absorbing region. As the illumination of the blue-absorbing maximum yielded an increased absorption of the green-absorbing maximum, we also measured the ultrafast dynamics of this reaction (Fig. S4). Here, we mainly observe a broad ESA signal between 450 and 600 nm and a GSB signal at 400 nm. Both signal decays can be described with several lifetimes and one infinity lifetime (extending beyond the 2-ns measurement window) and no detectable photoproduct formation (yet).

**Figure 5:**
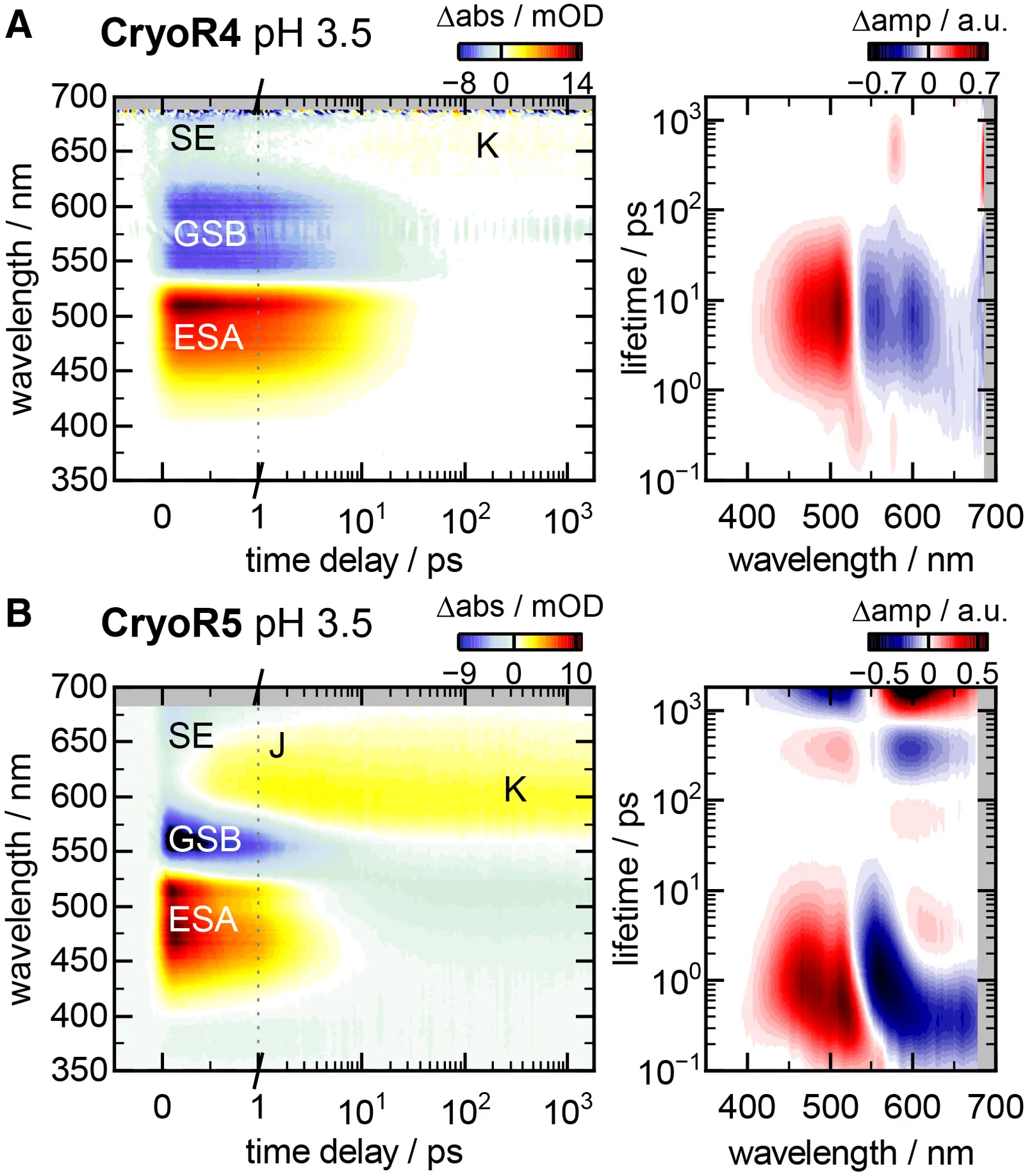
Ultrafast TA measurement of CryoR4 and CryoR5 at pH 3.5. (*A*) CryoR4 ultrafast map and LDM. (*B*) CryoR5 ultrafast map and LDM. The signal amplitude is color coded as follows: positive (*red*), none (*white*), and negative (blue) difference absorbance. The x-axis is linear until 1 ps and logarithmic afterward. The corresponding LDMs illustrate the kinetic components of the respective measurement.

Similar to the dark state spectra, only weak pH-induced effects were observed for the ultrafast dynamics of CryoR4 and CryoR5. Thus, we only show maps at pH 3.5 in the main text (Fig. 5), whereas maps at pH 8 can be found in the supporting material (Fig. S5). For CryoR4 at both pH values, a very strong, long-lived ESA decaying at ∼9 ps is observed, concurrently with most of the GSB signal. A very small fraction of the excited protein molecules transforms into the K intermediate, whereas the J state is not observed at all. The only detectable difference between pH 3.5 and pH 8 is a stronger SE at neutral pH. Overall, both measurements are almost identical and showcase the poor efficiency of the initial photoreaction of CryoR4, also underlined by the calculated GSB/ESA ratios between 1% and 3% (Table 1). This corresponds to approximately the same order of magnitude as the KR2 counterion mutant D116N mimicking the protonated RSB counterion. The dynamics observed for CryoR4, including a temporally elongated ES signal lifetime, the absence of the J intermediate, and a less efficiently populated K intermediate resemble many other known MRs, including CryoR1 and CryoR2, at acidic conditions (24,25,26,27,28). At higher pH values such as 8, an accelerated ES dynamic would be anticipated, as well as an efficient transition to more the J and K intermediates, which could not be observed here. Thus, the lifetimes and signal ratios for CryoR4 are almost completely pH independent.

For CryoR5 at the applied conditions, photon absorption leads to the formation of the ESA at 415–530 nm, the GSB at 540–590 nm, and the SE at 650–700 nm (Figs. 5 and S5). Although overall almost identical ultrafast dynamics are displayed, weak pH-dependent effects can be observed upon kinetic analysis of the data. At pH 8, the ES decays mono-exponentially with ∼0.3 ps leading to the formation of the J state, which subsequently relaxes at ∼1 ps to form the K intermediate. The results resemble the prototypical MR ultrafast dynamics at physiological pH. To follow the general trend of MRs, one would estimate an elongation for the ES lifetime, a loss of the J intermediate, and less K intermediate formation upon acidification (24,25,26,27,28). Surprisingly, this is not the case for CryoR5, where the only observable pH-induced effect is that the ES decays bi-exponentially. The main component of the ES decay still has a lifetime of ∼0.3 ps, whereas the remaining one decays temporally parallel to the J-to-K transition at ∼1 ps. This minor indication of a bi-exponential ES decay does not seem to result in a smaller fraction of the protein entering the photocycle, as corroborated by ESA/GSB ratios of 15% (pH 8) and 13% (pH 3.5), respectively. Therefore, both CryoR4 and CryoR5 appear to be almost pH independent on the ultrafast timescale, with opposing spectral signatures, as CryoR4 resembles low pH dynamics, whereas CryoR5 shows classical MR dynamics.

The ultrafast behavior of rhodopsins can generally be described by two models, which are heavily discussed in the field. One model is based on ES branching, the other on GS heterogeneity. The first model assumes a splitting of the ES population into a reactive and nonreactive pathway due to an energy barrier on the ES potential energy surface (PES) and branching at the conical intersection back to the GS. The PES and the energy barrier are influenced by the coupling between the S_1_ and S_2_ states, which is determined by the electrostatics of the RBP (24,25,45,46). This has been actually also supported by intensive modeling (47,48). The second model assumes GS heterogeneity in the dark state, which is modulated by the acid-base equilibrium of the counterion and the RBP hydrogen bonding network determining the population of the reactive and nonreactive pathway (27,49,50). Both models stress the general electrostatics in the RBP and the protonation state of the counterion as crucial factors in determining the initial reaction steps. Following the ES branching model, the fast dynamics and a comparatively large percentage of reactive photoproduct seen for CryoR2 at high pH and for CryoR5 would be attributed to a decreased energy for torsional motion around the C13–C14 double bond and a stabilized S_1_ PES with a lowered energy barrier, which favors the isomerization pathway (24,25,45,46). In line with the GS heterogeneity model, these ultrafast dynamics also point to a deprotonated counterion, as the negative charge can stabilize the S_1_ state, the positive charge of the retinal, decreases the double bond character and the energy required for torsional motion around the C13–C14 bond (27,47,49,50,51). The elongated dynamics for CryoR2 at low pH and CryoR4 are associated with an increased torsional motion energy. According to the ES branching model, an S_1_ PES, which is strongly modulated by coupling to the S_2_, therefore increases the energy barrier along the isomerization coordinate and elongates the ESA signal (24,25,45,46). This behavior hints to a protonated counterion, which in the GS heterogeneity picture mainly yields nonreactive S_1_ states (27,49,50). For CryoR4, the same reasons for pH independence as for CryoR5 can be stated, whereas CryoR2’s RBP electrostatics are changed by acidification, indicating the protonation of its counterion.

CryoR2 and CryoR3 both show the two-part ESA with a negative signal in the LDM at lower wavelengths indicating its rise, which should happen before the experimentally accessible timescale. The delayed rise of this positive signal might originate from its overlap with the GSB signal or vibrational cooling in the Franck-Condon region. A broad ESA as well as two different ESA regions suggest an altered PES modulated by the coupling of the respective S_1_ and S_2_, which could be further substantiated by theoretical calculations. The inhomogeneous behavior aligns with the absorption spectra of CryoR2 and CryoR3 showing the most changes upon pH variation, arguing that their RBPs are mostly accessible by the bulk, and suitable residues might be protonated or deprotonated upon pH changes.

Altogether, the four CryoRs enter a photocycle regardless of pH, partly with identical, partly with differing dynamics, and the respective K intermediates can be observed beyond the end of the ultrafast timescale of 1.8 ns.

#### CryoRhodopsins further diverge on slower timescales

The initial study on CryoRs at neutral to alkaline conditions implied a quite conserved behavior with regard to their photocycle kinetics on the microseconds to minutes timescale (3). Namely, all studied CryoRs displayed a long-lived M intermediate lasting several minutes (Fig. S6). The data presented in this paper show that CryoRs possess highly diverse light-induced characteristics at acidic pH conditions. Accordingly, here we investigated the photocycle dynamics of CryoR2–5 on the microsecond to minutes timescale at acidic conditions (pH 3.5).

Most known MRs show a modified or even no M signal upon acidification of the buffer, as it is becomes increasingly difficult to deprotonate the RSB, and the deprotonated RSB is easily reprotonated. At pH 5, NsXeR transitions back to the GS via a L_CP_ and L_EC_ intermediate with only a small percentage proceeding via M, whereas it shows to distinct M intermediates at pH 7 (35). In KR2, the M state is delayed, and the M-to-O transition is lost, as the counterion coordinates different residues instead of the RSB due to its protonation, and the sodium ion has to compete with the increased proton concentration (26). Even BR, which has a physiological pH of 4, loses its M signal when the pH is lowered further (52,53,54,55).

Upon acidification, CryoR2 loses some of its signal intensity, with the M intermediate completely decaying after more than 50 s. CryoR3 on the other hand displays a minutes-long M intermediate even at acidic pH, yet with four times less intensity. The M intermediate of CryoR4 is significantly shortened to seconds, the one of CryoR5 to milliseconds, whereas both retain a similar signal intensity.

**Figure 6:**
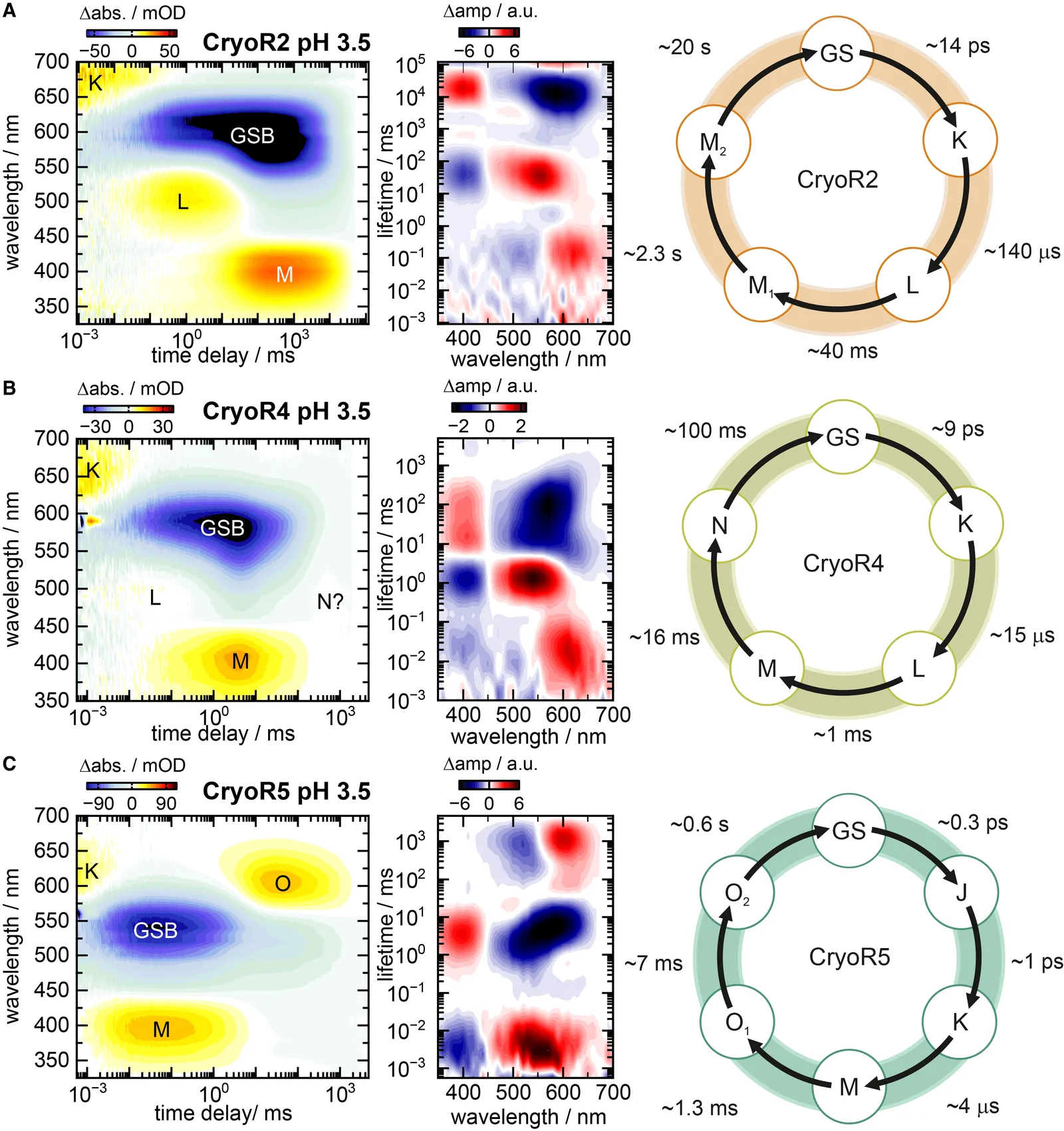
Flash photolysis measurements at pH 3.5 of CryoR2 (A), CryoR4 (B), and CryoR5 (C). The observed photocycle intermediates are named in the common way for microbial rhodopsins based on the photocycle of bacteriorhodopsin. In the middle, the corresponding LDMs are shown illustrating the kinetics components of the respective measurement. Additionally, photocycle schemes are shown for each CryoR.

Due to the long photocycle durations, it is not feasible to measure entire maps at pH 8 (and at pH 3.5 for CryoR3) as the signal is diminished by actinic effects caused by the probe light. However, the acceleration of the photocycles of CryoR2, CryoR4, and CryoR5 at acidic pH allows an examination over the whole wavelength range. Interestingly, they possess different intermediate states (Fig. 6), which is in line with their diverse ultrafast dynamics.

CryoR2 shows a signal at 650 nm belonging to the K intermediate, which is spectrally identical to the signal observed in the ultrafast measurements and transforms into an L intermediate in ∼140 μs. Then, the M state rises at ∼40 ms together with the decay of the L state. The near-UV-absorbing M state is still quite long-living and decays with a lifetime of ∼20 s. Such photocycle duration justifies observing accumulation of the M state in the steady-state experiments at pH 3.5 (Fig. 2). The M state consists of M_1_ and M_2_, with the transition around ∼2.3 s, which can be seen by the alternating rise and decay pattern with lower intensity between the M state rise and decay signals of the LDM (Fig. 6). For CryoR4, the positive signal at 650 nm belonging to the K intermediate decays at ∼15 μs, followed by the rise of a small positive signal at 490 nm corresponding to the L state. The L-to-M transition occurs at ∼1 ms, and the M intermediate decays within ∼16 ms. The lifetime of the M state is thus too short to observe its population in the steady-state illumination experiments (Fig. 2). The GSB decay maximum in the LDM is stretched until ∼100 ms, hinting at another late photocycle intermediate with a minimal positive signal around 480 nm at ∼100 ms, which we termed as the N state due to its temporal and spectral position. However, this state is only weakly populated. Usually the N state is more red-shifted than the L intermediate, overlapping strongly with the GSB signal (56,57,58,59). A similar blue shift of the N state signal has been recently observed for PR (28). An IR study could clarify the nature of the signal and determine the retinal configuration, but a hint is already given by the second bright state (SBS) signal at 330 nm, which is a marker band for 13-*cis* retinal (28) and does not decay until the end of N state, but due to overlap with the strong M state signal, no final statement can be made here (Fig. S7). The spectral structure observed for CryoR2 and CryoR4 can be compared with that of xenorhodopsins, which also display K, L, M, and an additional GS^∗^ intermediate not seen here (35). Both CryoR2 and CryoR4 have a visible L intermediate at acidic pH, indicating the accumulation of a precursor for the proton transfer reaction before the rise of the deprotonated species.

CryoR5, on the other hand, behaves differently from the other studied CryoRs at acidic conditions. First, it transforms directly from the K to the M intermediate already in the early microseconds without any L state signal. Afterward, a strong red-shifted positive signal at 610 nm rises together with the decay of M at ∼1.3 ms and is therefore termed the O intermediate. By comparing the transients of the SBS and the O intermediate (Fig. S7) and examining their lifetimes, two intermediates O_1_ and O_2_ with an O_1_-to-O_2_ transition at ∼7 ms could be identified. Both the O_2_ state and the GSB signals decay with a lifetime of ∼600 ms. As in the case of CryoR4, the short photocycle rationalizes the illumination experiments, where no decrease of the GS absorbance could be observed due to the photocycle being too fast to accumulate a population of the M state (Fig. 2). Since CryoR5 shares its three-letter motif with PR, a comparison is drawn here. At low pH, PR was reported to invert the vectoriality of its proton pumping, which is accompanied by a loss of the M intermediate (60), so CryoR5 might better be compared with higher pH measurements (61). However, besides the overall pattern, CryoR5 misses the N intermediate and possesses a much broader, stronger, and temporally long M. Therefore, the mechanism considerably differs and should not be compared.

Although the exact nature of each photointermediate is beyond the scope of this study, their usual role can be derived from BR (18,62). The L intermediate seen for CryoR2 and CryoR4 indicates prearrangement of the protein between the isomerization and the deprotonation step. This intermediate is not observed in CryoR5, which transitions directly from K to M. Reprotonation, reisomerization, and structural rearrangements happen concurrently for CryoR2, whereas CryoR4 has a small population of an assumed N intermediate, which still contains 13-*cis* retinal and is also associated with structural rearrangements, after which it also returns to the GS. CryoR5 on the other hand transitions to the GS via an O intermediate, which usually represents a non-GS protein structure with a reisomerized 13-*trans* retinal. As seen from the SBS comparison, O_1_ still contains 13-*trans* retinal, whereas the signal is absent for O_2_. Thus, reisomerization and structural rearrangements to the GS are temporally separated. The different photocycle schemes highlight the diversity in the CryoR subfamily and can either be caused by small structural changes or kinetic changes in the progression of isomerization, switching and transferring steps like that shown for NsXeR (35).

## Discussion

Although there are no experimental structural data on CryoR3–5 available, the data presented above allow for some presumptions concerning the molecular mechanisms underlying unusual spectroscopic properties of the proteins. Indeed, the results strongly suggest that the ways of forming this near-UV-absorbing state and, in particular, the proton acceptor from the RSB upon formation of the M state, vary within the clade. In most cases, such as for CryoR1, CryoR2, CryoR3, and CryoR5, we conclude that carboxylic residues, either aspartate or glutamate or their complex, depending on the functional motif of the protein, act as a proton acceptor group from the RSB.

The absorption maxima of the CryoRs are located between 490 and 590 nm (neutral pH) and 630 nm (acidic pH). The spectral position of the MR absorption maxima highly depends on a variety of factors, including electrostatic effects, protonation status of and distances between residues, as well as variations in hydrogen bonding, which also has been confirmed and supported by theoretical work (63,64). Especially the interaction with the counterion has been shown to possess a major influence (65,66,67). We cannot pinpoint the determinants on a molecular level with certainty, but based on our observation that CryoR3-5 do not exhibit spectral wavelength shifts upon pH change, we suspect that the protonation state of their respective counterion(s) remains the same across a wide pH range. It is likely that these effects are caused by variations in the hydrogen bonding network of the motif residues, which we cannot analyze further without structural data. The key role of hydrogen-bonded networks for proton transfer steps has also been shown by computational studies (68), as hydrogen bond networks contribute to the determination of proton transfer pathways and to the stability of transient photocycle intermediates.

**Figure 7:**
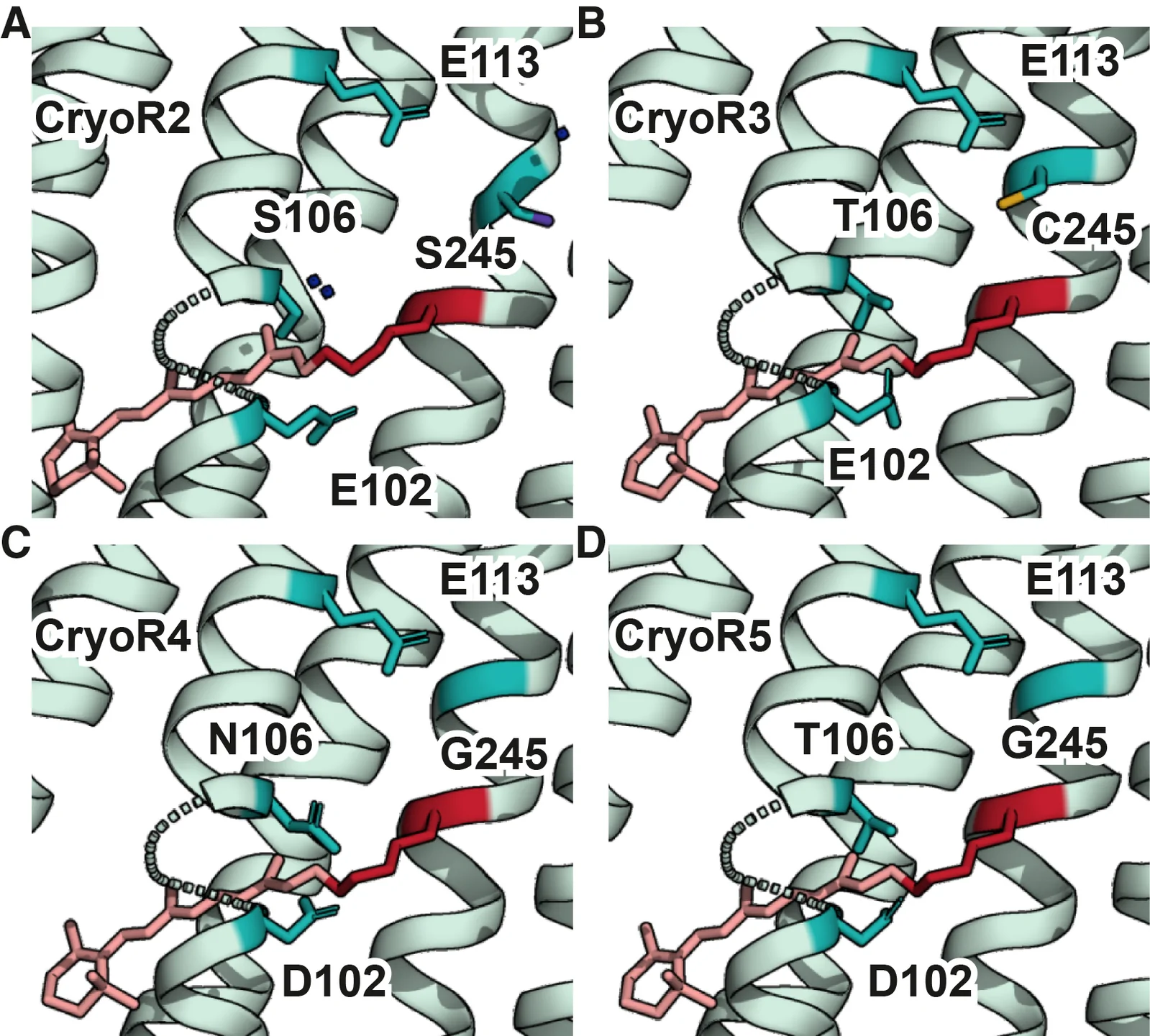
Structures of CryoR2-5 including the X245 position. CryoR2 (A) and CryoR3 (B) contain Ser or Cys, respectively, whose heteroatoms were colored for better distinction. CryoR4 (C) and CryoR5 (D) both contain Gly at position 245, taking up less space. Motif, retinal, and K241 are colored as in Fig. 1.

For CryoR1 and CryoR2, the counterion complex becomes protonated at acidic pH, notably delaying the formation of the M state. For CryoR3 and CryoR5, absorption spectra and ultrafast kinetics suggest that the counterion(s) remain deprotonated in the wide range of pH, allowing for effective formation of the M state. However, the lifetime of the M state in CryoR5 is significantly shortened at low pH, and the strongly red-shifted O state is formed, indicating that the RSB is quickly reprotonated in this protein under acidic conditions with the counterion still being protonated. Thus, we suggest that in CryoR5, the RSB reprotonation at high proton concentration proceeds fast from the cytoplasmic side. This is surprisingly not the case for CryoR3, where the RSB is not reprotonated until the protein returns to the GS in minutes even at pH as low as 3.5, meaning that the proton is likely transferred back from the counterion to the RSB after thermal retinal reisomerization back to the all-*trans* configuration. From the amino acid sequence analysis, we suggest two possible explanations for this difference: 1) C245 is found in CryoR3 (Fig. 7) in the region of putative proton transfer between the cytoplasm and the RSB, which is substituted with glycine (G245) in CryoR5, thus offering more space for proton transport to reprotonate the RSB, and 2) the counterion complex of CryoR3 is composed of E102/D237 residues, which, by structural homology to CryoR1 and CryoR2, might share the proton and temporarily store it close to the RSB, thus lowering the pKa of the base in the M state, whereas in CryoR5 the counterion complex is D102/D237 and likely involves a water molecule mediating their interactions, with smaller effects on the pKa value of the RSB. CryoR4 represents a unique functional motif RR DNE DK, also coupled to its unusual spectral properties. Our cumulative data allow us to suggest that the counterion complex of CryoR4 is protonated in pH range from 3.5 to 10.5. The alternative hypothesis is that the presence of asparagine residue (N106) in a very close proximity to the RSB of CryoR4 results in a completely different configuration of the central region of the protein than that of other studied CryoRs, which likely remains independent of pH. Nevertheless, the RSB deprotonation occurs in CryoR4 at pH 3.5–10.5, suggesting proton transfer from the RSB toward the cytoplasmic side. A similar proton transfer pathway has been proposed for CryoR1 with a partially protonated counterion complex, resulting in weak net inward proton transport activity (3). We also cannot exclude that the same proton transfer occurs in CryoR2 under acidic conditions. Indeed, CryoR1, CryoR2, and CryoR4 at low pH share a similar spectral feature of their photocycles, such as the presence of the L state, not found in CryoR3 and CryoR5. The usual role of the L photointermediate can be derived from the data available on BR and xenorhodopsins (18,35,36,62). The L intermediate typically indicates prearrangement of the protein between the isomerization and the RSB deprotonation step and involves the arrangement of hydrogen bond network at the cytoplasmic internal part of rhodopsins. Thus, the presence of the L state might suggest that the mechanism of proton transfer from the RSB toward the cytoplasm also involves similar structural changes in CryoRs.

## Conclusion

The UV/vis photophysical properties of CryoR2, CryoR3, CryoR4, and CryoR5 were elucidated from femtoseconds to minutes, and their distinctiveness in the MR family is presented. Although typically rhodopsins from the same clade show similar patterns in their behavior, each CryoR has individual features. CryoR2 is similar to CryoR1 in its wavelength shift and ultrafast pH dependence but has a completely different photocycle. CryoR3 generally already has a high share of deprotonated retinal in the dark state and is the only CryoR to retain the slow M at acidic pH. The spectral features in the ultrafast are responsive to pH changes, but the signal intensity stays weak throughout the entire investigated pH range. Its unique feature is the ability to increase the absorbance of the main peak by blue light illumination. CryoR5 and CryoR4 both show an extraordinary pH independence consistent across ultrafast and steady-state data, but with completely different efficiency and contrasting photocycles. Although CryoR5’s ultrafast behavior corresponds to a fast photoreaction favoring the isomerization pathway, CryoR4’s dynamics are much slower. Beyond a shortened M, they do not share any common photointermediates on the microsecond to minute timescale.

In summary, we demonstrate that the RSB deprotonation in CryoRs, required for the formation of the extremely long-living near-UV-absorbing state, can proceed in different ways for various proteins of the clade. Nevertheless, the dominant near-UV-absorbing state at neutral and alkaline pH is the only spectral feature preserved in all studied CryoRs, further pointing out its functional relevance. Structural studies have to be performed to reveal the exact molecular mechanism behind the differing photocycles.

## Data and code availability

All data sets for this article are available at Goethe University Data Repository (GUDe) doi: 10.25716/gude.1mj1-4baa.

## Author contributions

S.W., G.H.U.L., and J.W. conceived and designed the experiments. S.W. and G.H.U.L. performed the experiments and analyzed the data. K.K. supplied the samples and contributed to discussion. S.W. wrote the manuscript, which was reviewed and edited by G.H.U.L., K.K., and J.W.

**Funding**

This research was supported by the German Research Foundation (CRC 1507–Membrane-associated Protein Assemblies, Machineries and Supercomplexes; project 05 to J.W.). K.K. has been supported by EMBL interdisciplinary Postdoctoral Fellowship (EIPOD4) under Marie Sklodowska-Curie Actions cofund (grant agreement number 847543).

## Declaration of interests

The authors declare no competing interests.

## Supporting Material

Document S1. Figures S1–S7 and Table S1

Document S2. Article plus Supporting Material
