# Supplementary material for: Diverging pH dependence and photocycle dynamics across members of the CryoRhodopsin clade
Source: Biophys J. 2026 Jan 23;125(10):2343–55. doi: 10.1016/j.bpj.2026.01.031 (PMC13351561; doi:10.1016/j.bpj.2026.01.031)
Supplement: Supplementary file 1 [file mmc1.pdf]

Biophysical Journal, Volume *125*

Supplemental information

Diverging pH dependence and photocycle dynamics across members of the CryoRhodopsin clade

Sarah Warrelmann, Gerrit H.U. Lamm, Kirill Kovalev, and Josef Wachtveitl

SUPPLEMENTAL MATERIAL

An online supplement to this article can be found by visiting BJ Online at http://www.biophysj.org.

	46	82	85	89	96	212	216
**BR**	T	R	D	T	D	D	K
	57	99	102	106	113	237	241
**CryoR1**	R	R	E	A	E	D	K
**CryoR2**	R	R	E	S	E	D	K
**CryoR3**	R	R	E	T	E	D	K
**CryoR4**	R	R	D	N	E	D	K
**CryoR5**	R	R	D	T	E	D	K
**DSE009**	S	R	D	S	E	D	K
**DSE307**	S	R	D	S	E	D	K
**DSE248**	N	R	D	S	E	D	K

Figure S1: **Comparison of motifs.** The amino acids of the seven-letter motifs of BR, CryoR1-5 and phylogenetically near DSE rhodopsins are shown. For easier recognition BR numbering as well as CryoR1 numbering are used.

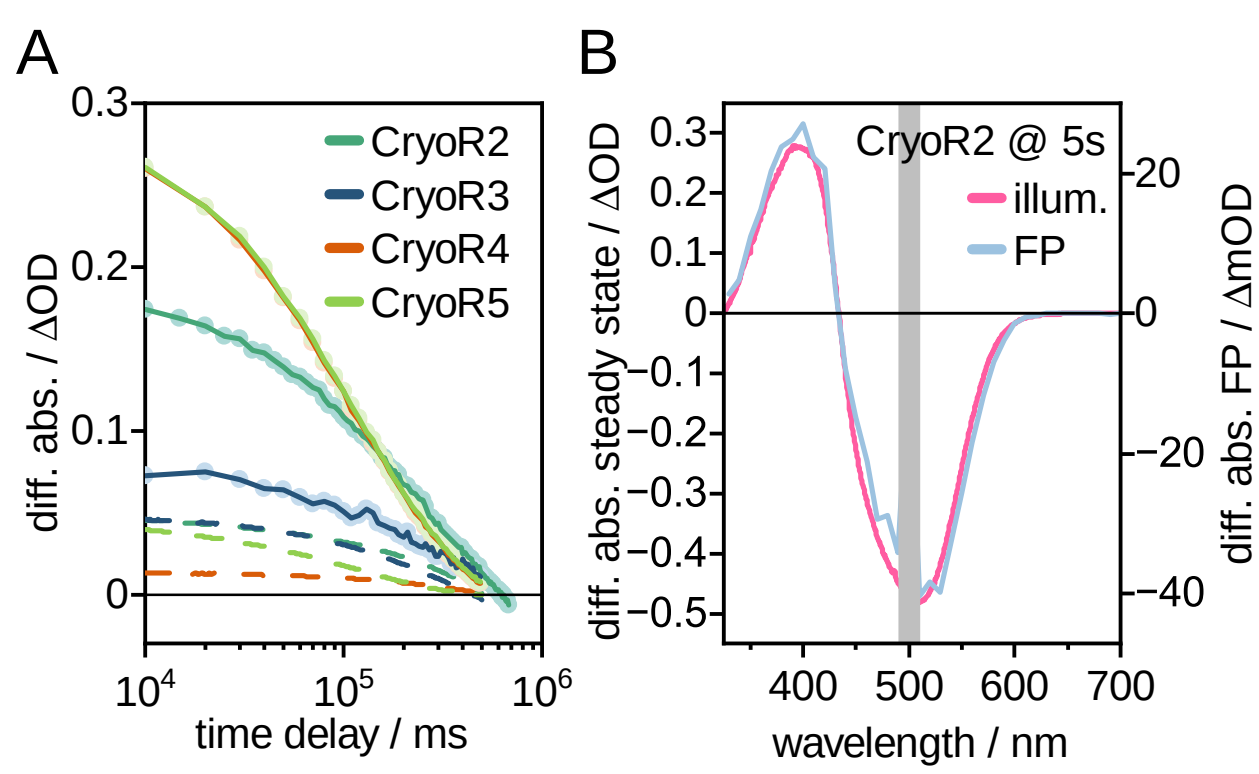

Figure S2: **Comparison between steady state illumination and flash photolysis data.** A) Decay signals at 400 nm after illumination with a LED (solid lines) and after excitation in the flash photolysis setup (dashed lines). With both methods, the decay occurs at approximately the same rate. (B) Difference spectrum of CryoR2 5 s after illumination into the PSS in steady state and after excitation in the flash photolysis setup. Intensity discrepancies are due to differences in the setups.

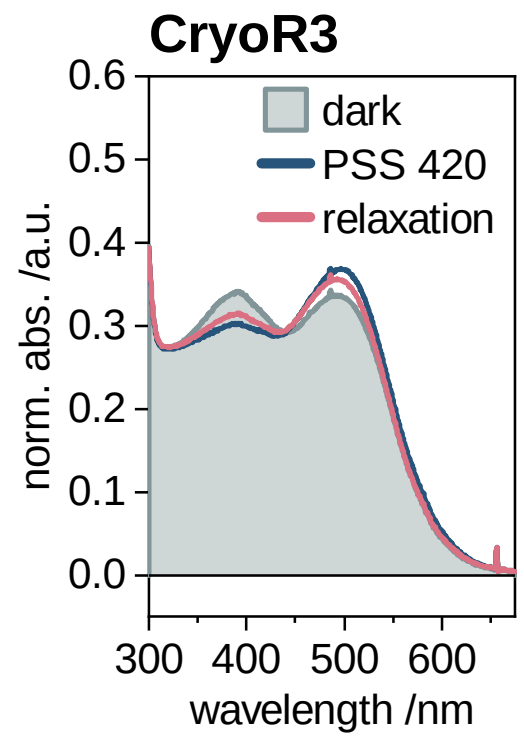

Figure S3: **Switching behaviour of CryoR3 at pH 8 after excitation with 420 nm LED.** The cuvette was prepared in the dark and additionally kept in the dark for 2 h before the experiment. Illumination leads to an increase of the 490 nm maximum. In darkness, the sample relaxed back to the ground state. The shown relaxation was recorded after 100 s.

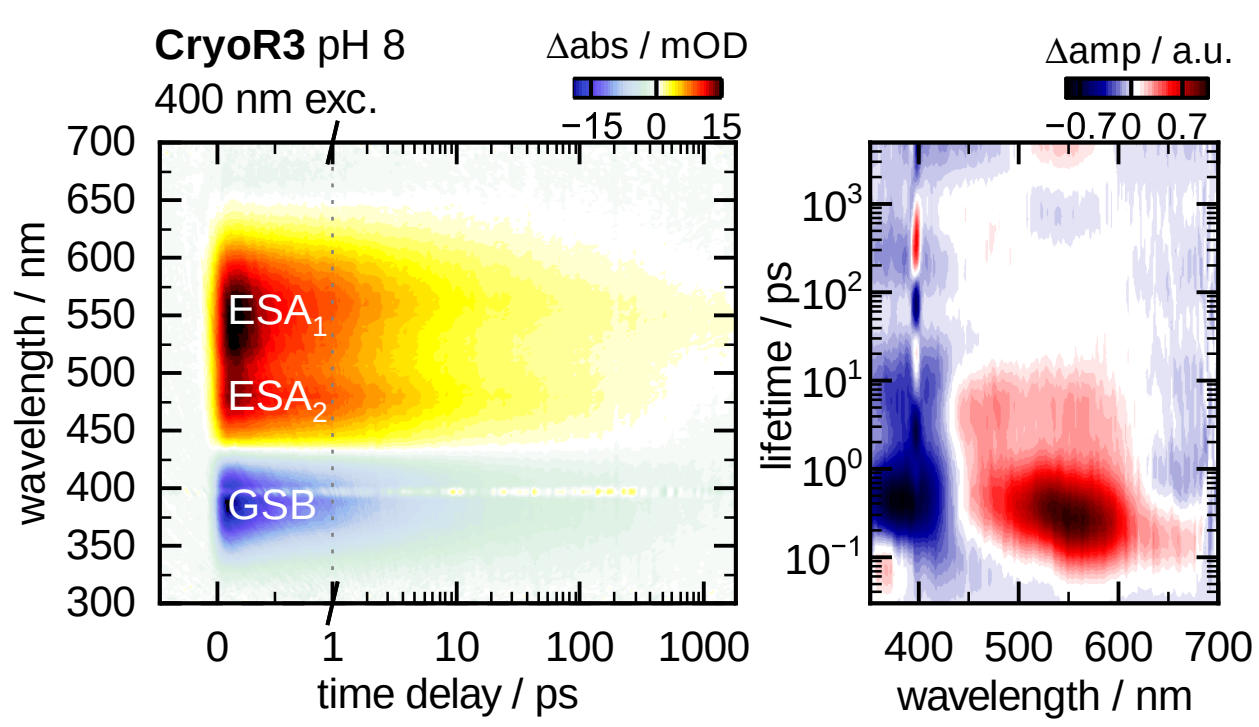

Figure S4: **Ultrafast TA measurement of CryoR3 excited at 400 nm at pH 8.** CryoR3 ultrafast map (left) and LDM (right) with excitation into the blue absorbing maximum at 400 nm. The signal amplitude is color coded as follows: positive (red), none (white) and negative (blue) difference absorbance. The x-axis is linear until 1 ps and logarithmic afterwards. The corresponding LDMs illustrate the kinetic components of the respective measurement.

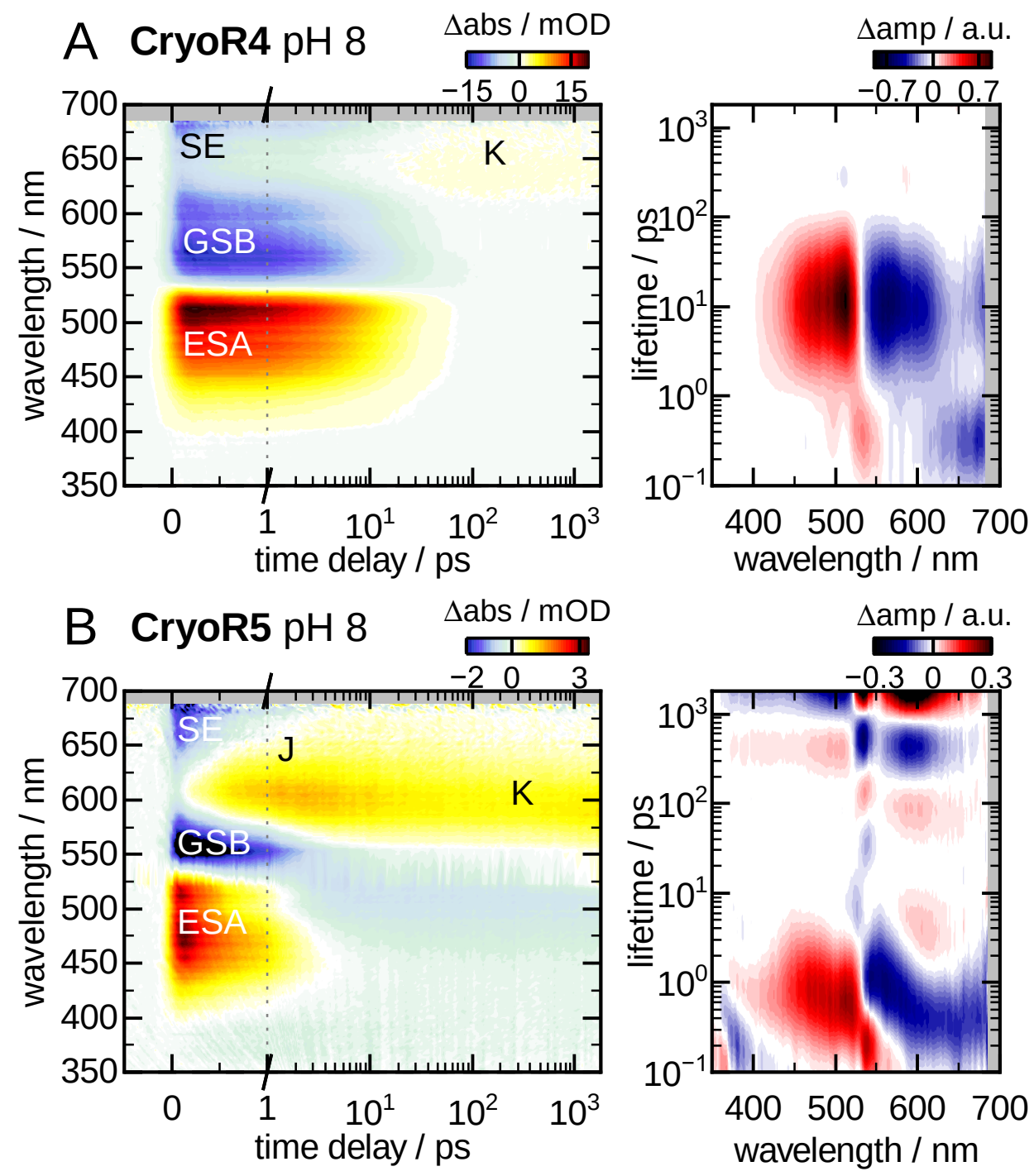

Figure S5: **Ultrafast TA measurement of CryoR4 and CryoR5 at pH 8.** (A) CryoR4 ultrafast map and LDM. (B) CryoR5 ultrafast map and LDM. The signal amplitude is color coded as follows: positive (red), none (white) and negative (blue) difference absorbance. The x-axis is linear until 1 ps and logarithmic afterwards. The corresponding LDMs illustrate the kinetic components of the respective measurement.

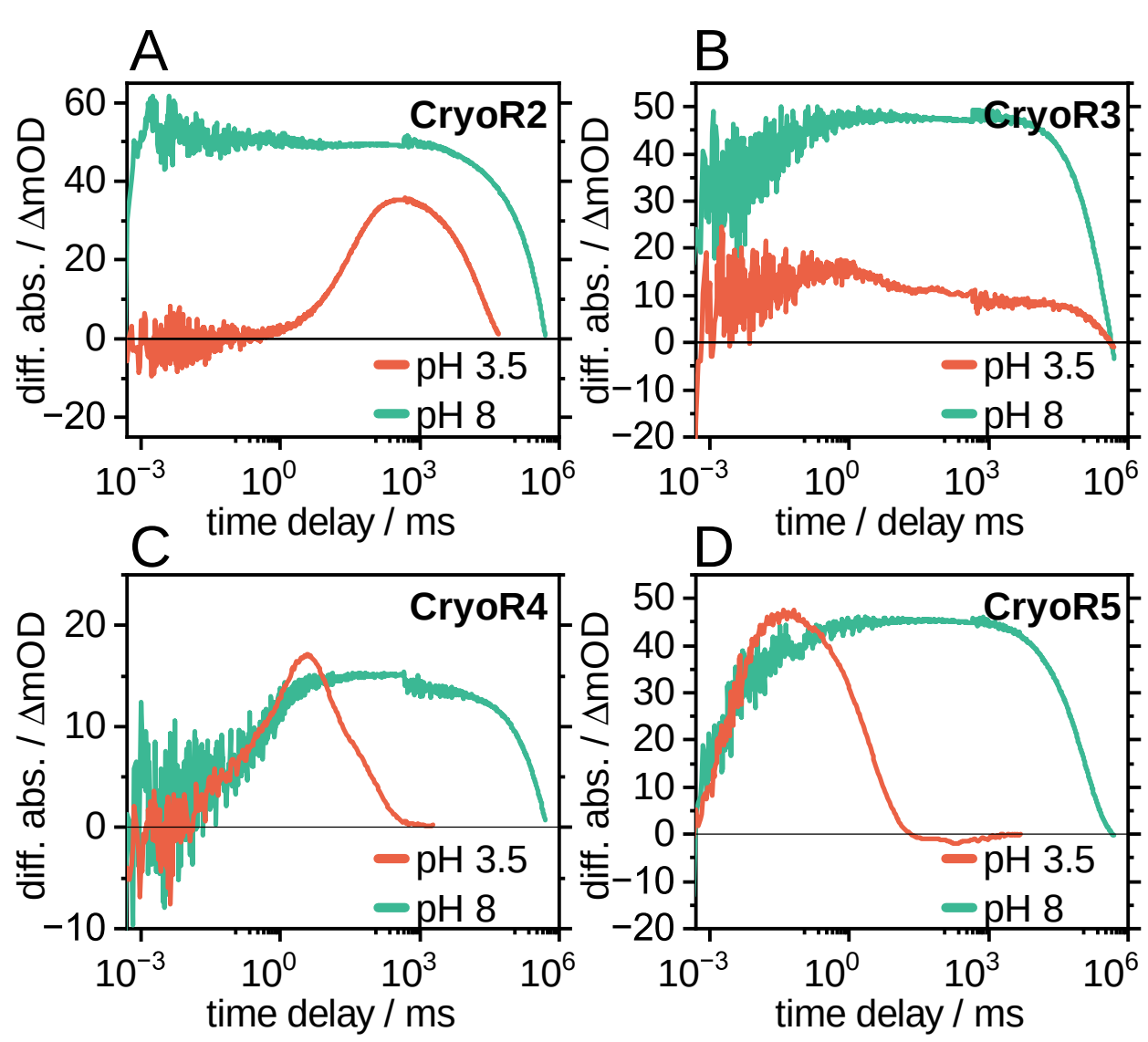

Figure S6: **Transients at 400 nm for all CryoRs.** Transients at 400 nm of CryoR2 (A), CryoR3 (B), CryoR4 (C) and CryoR5 (D) were newly recorded at pH 3.5 (red line). Transients at pH 8 (green line) were taken from Lamm et al.(3). The logarithmic timescale stretches to several minutes.

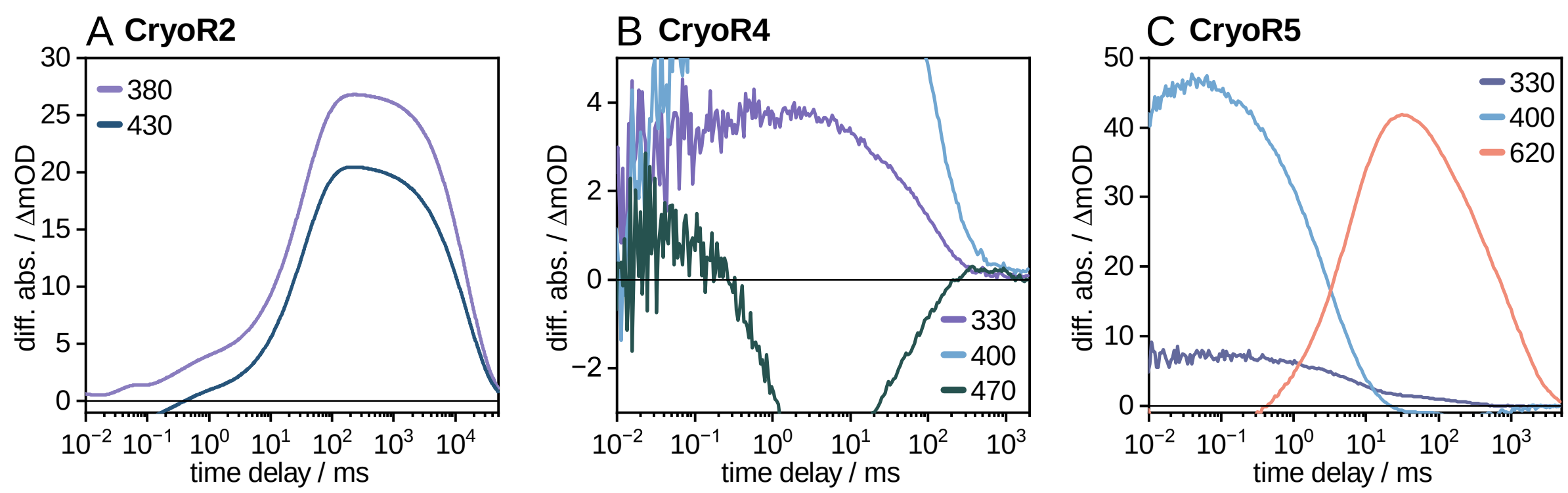

Figure S7: **Transients at different wavelengths for CryoR2, CryoR4 and CryoR5 demonstrating the discussed intermediates.** (A) The transient at 380 nm represents M_1, and the transient at 430 nm shows the later rising M_2 of CryoR2. (B) The transients represent the SBS (330 nm), M (400 nm) and N (470 nm) of CryoR4. The SBS and N decay similarly, however the signals are superimposed by the strong M-signal. (C) The transients each represent the SBS (330 nm), M (400 nm) and O (620 nm) of CryoR5. The SBS decays before the O_2 signal.

	GSB signal / mOD	ESA signal / mOD	ratio
**CryoR5 pH 3.5**	1.0	7.7	13%
**CryoR5 pH 8**	0.4	2.7	15%
**CryoR4 pH 3.5**	0.5	13.2	3%
**CryoR4 pH 8**	0.2	16.5	1%
**CryoR3 pH 3.5**	0.0	1.2	2%
**CryoR3 pH 8**	0.2	4.9	5%
**CryoR2 pH 3.5**	0.2	10.0	2%
**CryoR2 pH 10.5**	2.4	9.4	26%
**KR2 pH 5.5**	1.8	15.3	12%
**KR2 pH 9**	3.0	6.8	45%
**KR2 D116N pH 9**	0.7	21.1	3%

Table S1: **Approximated ultrafast efficiency ratios for each CryoR.** The pH dependent ratios were calculated by dividing the value of the remaining GSB after 500 ps by the initial ESA signal at 80 fs. Transients for KR2 were taken from Asido et al.(23)
